# Neonatal anesthesia exposure impacts brain microRNAs and their associated neurodevelopmental processes

**DOI:** 10.1038/s41598-018-28874-0

**Published:** 2018-07-13

**Authors:** Daisy Lin, Jinyang Liu, Zihua Hu, James E. Cottrell, Ira S. Kass

**Affiliations:** 1 Anesthesiology Department, State University of New York Downstate Medical Center, Brooklyn, New York, USA; 2 The Robert F. Furchgott Center for Neural and Behavioral Sciences, Department of Physiology and Pharmacology, State University of New York Downstate Medical Center, Brooklyn, New York, USA; 3 Center for Computational Research, New York State Center of Excellence in Bioinformatics & Life Sciences, State University of New York at Buffalo, Buffalo, USA

## Abstract

MicroRNAs (miRNAs), when subjected to environmental stimuli, can exhibit differential expression. As critical regulators of gene expression, differential miRNA expression has been implicated in numerous disorders of the nervous system. In this study, we focused on the effect of a general anesthetic, as an environmental stimulus, on miRNA expression of the developing brain. General anesthetics have potential long-lasting neurotoxic effects on the developing brain, resulting in behavioral changes in adulthood. We first carried out an unbiased profiling approach to examine the effect of single-episode neonatal general anesthetic, sevoflurance (sevo), exposure on miRNA expression of the brain. Neonatal sevo has a significant effect on the expression of specific miRNAs of the whole brain and the hippocampus that is both immediate – directly after neonatal treatment, as well as long-lasting - during adulthood. Functionally, neonatal sevo-associated miRNA gene-targets share potential neurodevelopmental pathways related to axon guidance, DNA transcription, protein phosphorylation and nervous system development. Our understanding on the role of miRNAs provides a putative epigenetic/molecular bridge that links neonatal general anesthetic’s effect with its associated functional change.

## Introduction

In the brain, regulation of gene expression is orchestrated in a spatiotemporal manner to ensure proper development and function of neurons. microRNAs (miRNAs) have been recognized to fulfill this regulatory role by fine-tuning gene expression in the intricate network of neurons. miRNAs are endogenously expressed, ~21 bp small noncoding RNAs and have been found in plants and all animals^1,2^. However, miRNAs are subject to various environmental stimuli, resulting in their differential expression^3–8^. Neonatal exposure to anesthetics as an environmental stimuli, is neurotoxic to the developing brain^9^. Although numerous studies have shown long-lasting cellular and behavioral changes as a result of neonatal anesthetic exposure, the underlying epigenetic/molecular mechanism, specifically, the role of miRNAs is unknown. For this study, we conducted an unbiased investigation on the role of brain miRNAs as a result of neonatal sevoflurane (sevo) exposure, one of the most commonly used volatile anesthetics for pediatric patients^10^.

miRNAs are abundantly expressed in the brain and are critical regulators of gene expression by pairing with protein coding mRNA to initiate repression of translation or mRNA degradation^11–13^. At the cellular level, expression of miRNA is critical for the survival of not only differentiating, but also mature neurons. At the functional level, differential miRNA expression has been implicated in numerous neuropsychological and neurological disorders such as, autism spectrum disorder^14^, schizophrenia^15,16^, Huntington’s disease^17^, Alzheimer’s disease^18^, addiction^19^ and Down syndrome^20^.

Neonatal anesthesia has consistently been associated with neuronal damage such as increased apoptosis, impaired dendritic and axonal branching and altered neurogenesis^21–25^. Equally consistent is its association with behavioral changes later on in life, including work from our lab and some human studies that showed decreased social interaction, impaired learning and memory and increased anxiety-like behaviors^22,26,27^. The current study is a direct follow-up that examines the role of miRNAs in neonatal anesthetic neurotoxicity. In addition to the overview on the expression pattern of brain miRNAs, we learned that neonatal sevo has immediate and long-lasting effects on the expression of specific miRNAs. Bioinformatics analysis provides insightful understanding of the functional relevance of these miRNAs on the neurodevelopmental process and gene expression regulation. Our data provides a putative epigenetic/molecular bridge that links neonatal sevo’s effect with the observed functional outcome at the behavioral level.

## Results

### Profiling of brain miRNAs from the P7-P7 group

**Figure 1:**
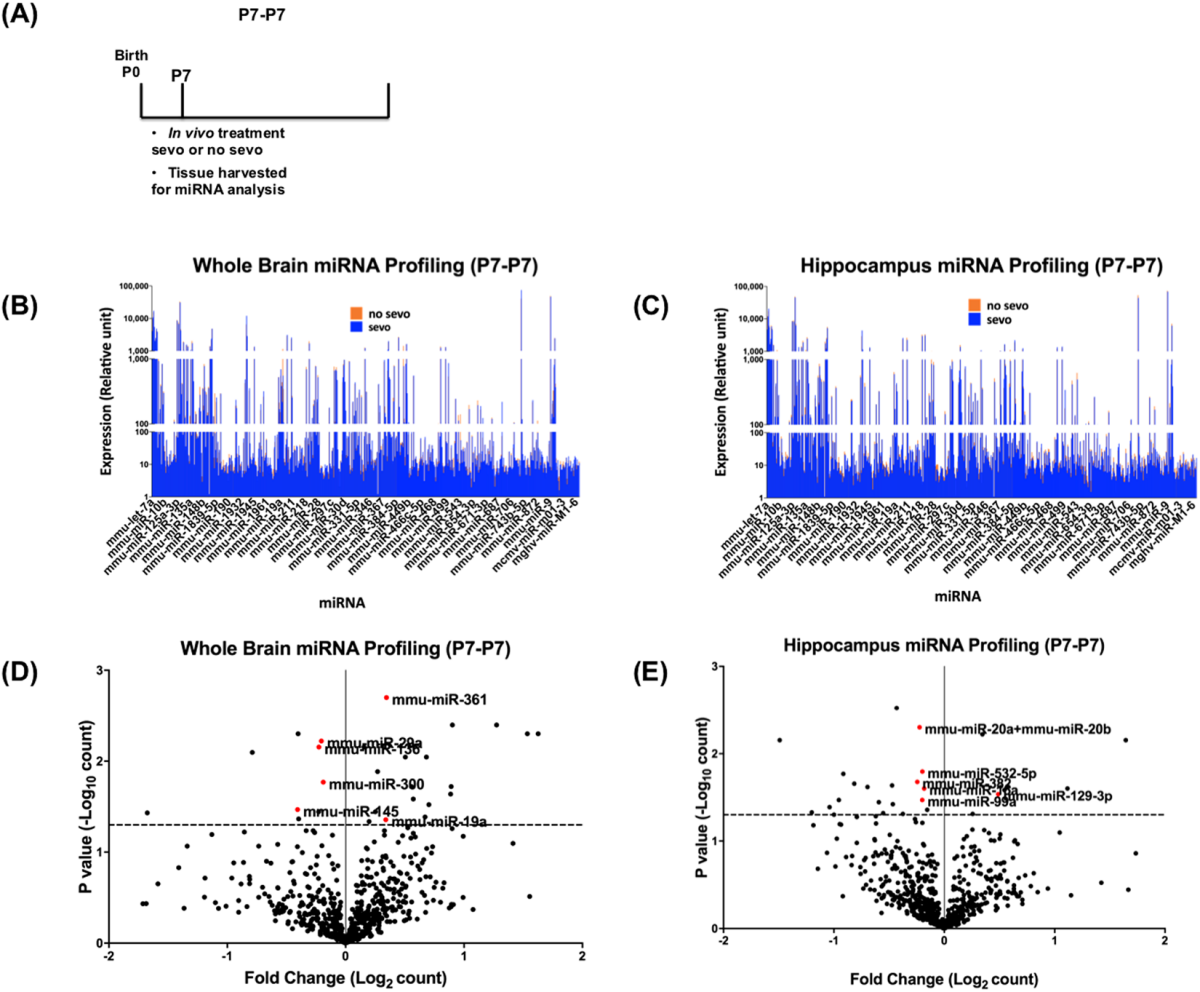
Profiling of miRNA expression after neonatal sevo treatment (P7-P7). (**A**) The P7-P7 group represents mice that were treated at P7 followed directly by tissue collection at P7. Profiling of the 599 known mouse miRNAs from the whole brain (**B**) and the hippocampus (**C**) yielded highly heterogeneous expression patterns. Neonatal sevo did not have a global effect on miRNA expression, comparing all miRNAs in the profiling data between the no sevo vs. sevo groups (Two-way ANOVA, (I) treatment: no sevo vs. sevo, P = 0.34 for the whole brain, P = 0.23 for the hippocampus; (II) miRNAs: comparing across miRNAs, P < 0.0001 for the whole brain and the hippocampus). (**D** and **E**) Volcano plots of the profiling data show both the fold change as a result of P7 sevo treatment and miRNAs that are differentially expressed with P < 0.05 (above the dotted line). Only miRNAs (red dots) with P < 0.05 and expression over 100 (relative unit defined by the Nanostring Technology) are selected to be in Table 1 for further analysis (Multiple t-tests).

In order to understand neonatal sevo’s immediate impact on miRNA expression, brain tissue was harvested directly after treatment and miRNA expression profiling was performed for this P7-P7 group (Fig. 1A). Both the whole brain and the hippocampus showed heterogeneous miRNA expression patterns. Neonatal sevo treatment on the P7-P7 group, did not affect miRNA expression globally (as a group), comparing all the miRNAs in the no sevo group vs. all the miRNAs in the sevo group (Fig. 1B, C) (Two-way ANOVA, (I) treatment, no sevo vs. sevo, P = 0.34 for the whole brain, P = 0.23 for the hippocampus; (II) miRNAs, all 599 miRNAs, P < 0.0001for the whole brain and the hippocampus, (III) interaction, interaction between treatment and miRNA, P > 0.999 for the whole brain and the hippocampus). Data show that P7 sevo treatment did not influence miRNA expression as a group and the difference in the expression of miRNAs is not related to the effect of the treatment.

While neonatal sevo had no global impact on brain miRNA expression in the P7-P7 group, significant change in the expression is shown for specific miRNAs (Fig. 1D,E) (multiple t-tests, P < 0.05). The list of differentially expressed miRNAs identified from the profiling analysis is shown in Table 1.

**Table 1:** Differentially expressed miRNAs as a result of P7 sevo treatment identified from the profiling list.

Differentially expressed miRNA	P value	Fold-change due to P7 sevo treatment
Whole Brain, P7-P7		
miR-136	0.03	−1.17
miR-145	0.03	−1.33
miR-19a	0.05	1.26
miR-29a	0.02	−1.15
miR-300	0.02	−1.14
miR-361	0.004	1.27

Putative gene targets		
2866		
Hippocampus, P7-P7		
miR-129-3p	0.05	1.4
miR-15a	0.04	−1.14
miR-20a+20b	0.02	−1.17
miR-382	0.03	−1.18
miR-532-5p	0.02	−1.15
miR-99a	0.04	−1.15

Putative gene targets		
2410		

**Figure 2:**
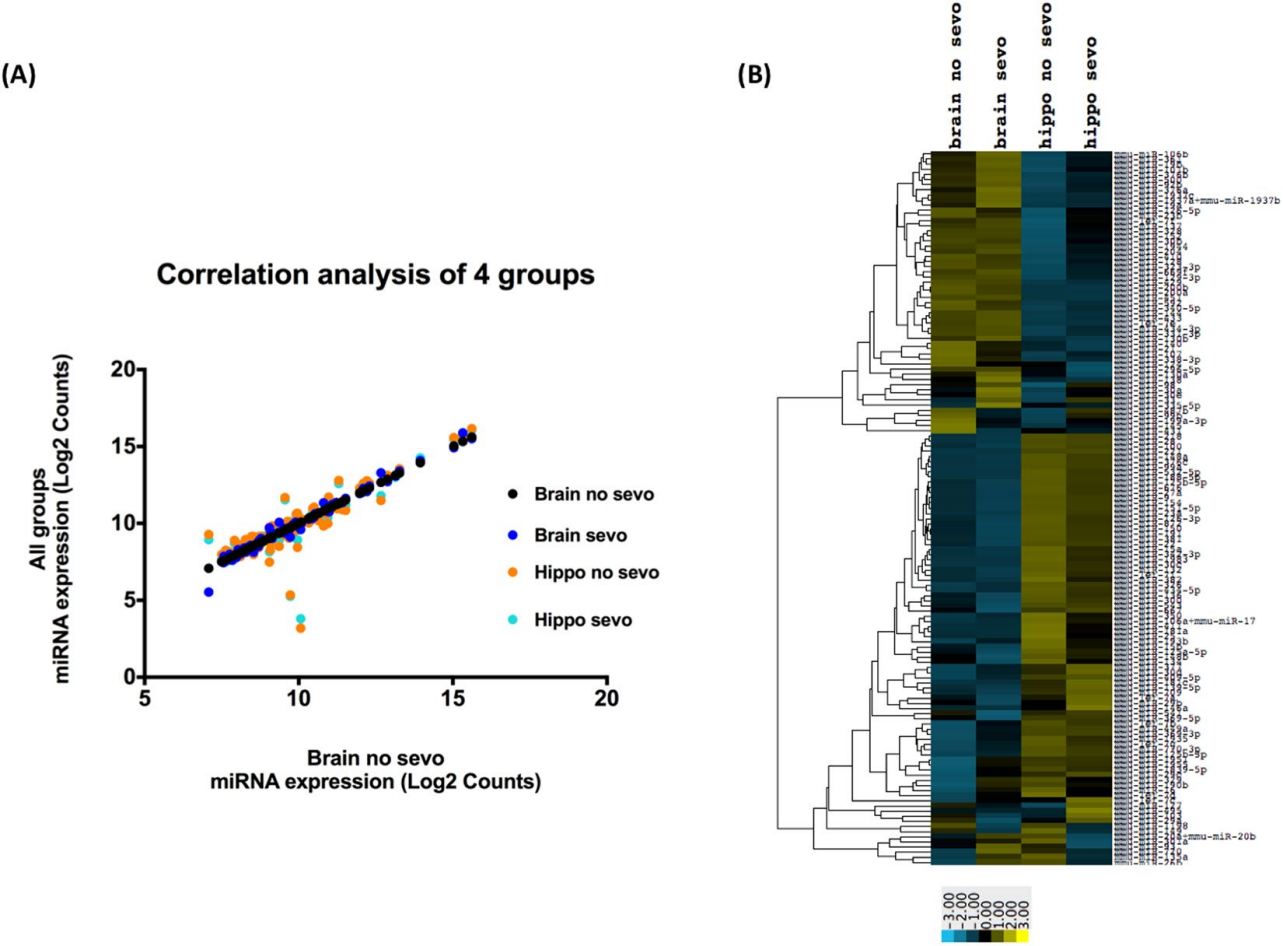
Correlation analysis of miRNA profiling data (P7-P7). (**A**) The plot is based on whole brain no sevo’s correlation to the other 3 groups, brain sevo, hippo no sevo, hippo sevo. (**B**) Heatmap analysis on the 4 groups shows that the expression pattern remained similar for sevo and no sevo within the whole brain and the hippocampus. However, between regions, the color bar shifted from higher expression (yellow) in the whole brain groups to lower expression (blue) in the hippocampus groups in the top half of the heatmap. This pattern was then reversed in the lower half of the heat map, with lower expression in the whole brain groups and higher expression in the hippocampus groups.

Correlational and heatmap analysis were conducted based on the miRNA profiling data to understand whether there is a global difference in miRNA expression when comparing the whole brain vs. the hippocampus. Such analysis would provide information on whether there is a brain region specific expression pattern of miRNAs. First, correlations of the four groups of miRNAs are plotted (Fig. 2A). Similar to the data from Fig. 1B and C, we observed that globally, neonatal sevo treatment did not impact miRNA expression patterns, such that the correlations between no sevo vs. sevo are significant (Table 2) (P < 0.0001). We then compared the whole brain vs. the hippocampus and observed that although correlations are also significant (Table 3) (P < 0.0001), the R^2^ values are lower for all groups of whole brain vs. hippocampus (Table 3) compared to all groups of no sevo vs. sevo (Table 2). A more apparent difference between the whole brain and the hippocampus is observed in the heatmap analysis (Fig. 2B). miRNAs that showed higher expression (yellow hue) in the whole brain than the hippocampus are clustered on the top half of the heatmap. miRNAs that showed lower expression (blue hue) in the whole brain compared the hippocampus is clustered in the bottom half of the heatmap. Data suggest hippocampus miRNAs have a region specific expression pattern compared to the whole brain.

**Table 2:** Correlational analysis between no sevo vs. sevo groups.

no sevo vs. sevo	Brain no sevo vs. Brain sevo	Hippo no sevo vs. Hippo sevo
R^2^	0.99	1

**Table 3:** Correlational analysis between the brain and the hippocampus groups.

Brain vs. Hippo	Brain no sevo vs. Hippo no sevo	Brain no sevo vs. Hippo sevo	Brain sevo vs. Hippo no sevo	Brain sevo vs. Hippo sevo
R^2^	0.87	0.89	0.86	0.88

### Putative functional pathways of sevo-associated miRNA gene-targets

**Figure 3:**
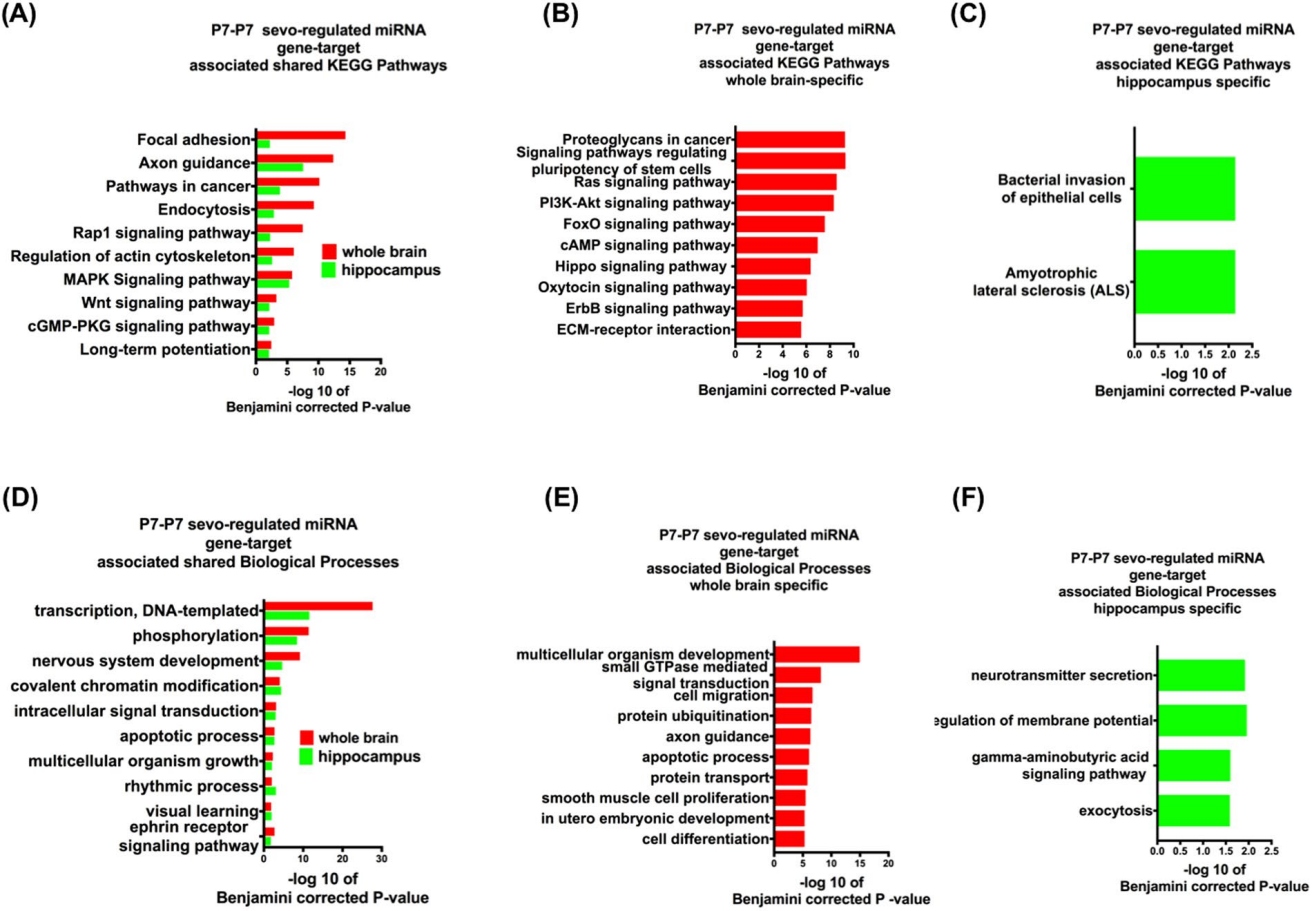
P7-P7 sevo-regulated miRNA gene-target associated KEGG pathways and Biological processes. P7-P7 sevo-regulated miRNA (from Table 1, both up and down-regulated miRNAs) gene-targets have shared (**A** and **D**), whole brain specific (**B** and **E**) and hippocampus specific (**C** and **F**) KEGG pathways and biological processes. (Pathways shown are based on Benjamini corrected p-value of <0.05, with only the top ten pathways shown. See Supplementary Figures for additional pathways within the analysis.).

**Figure 4:**
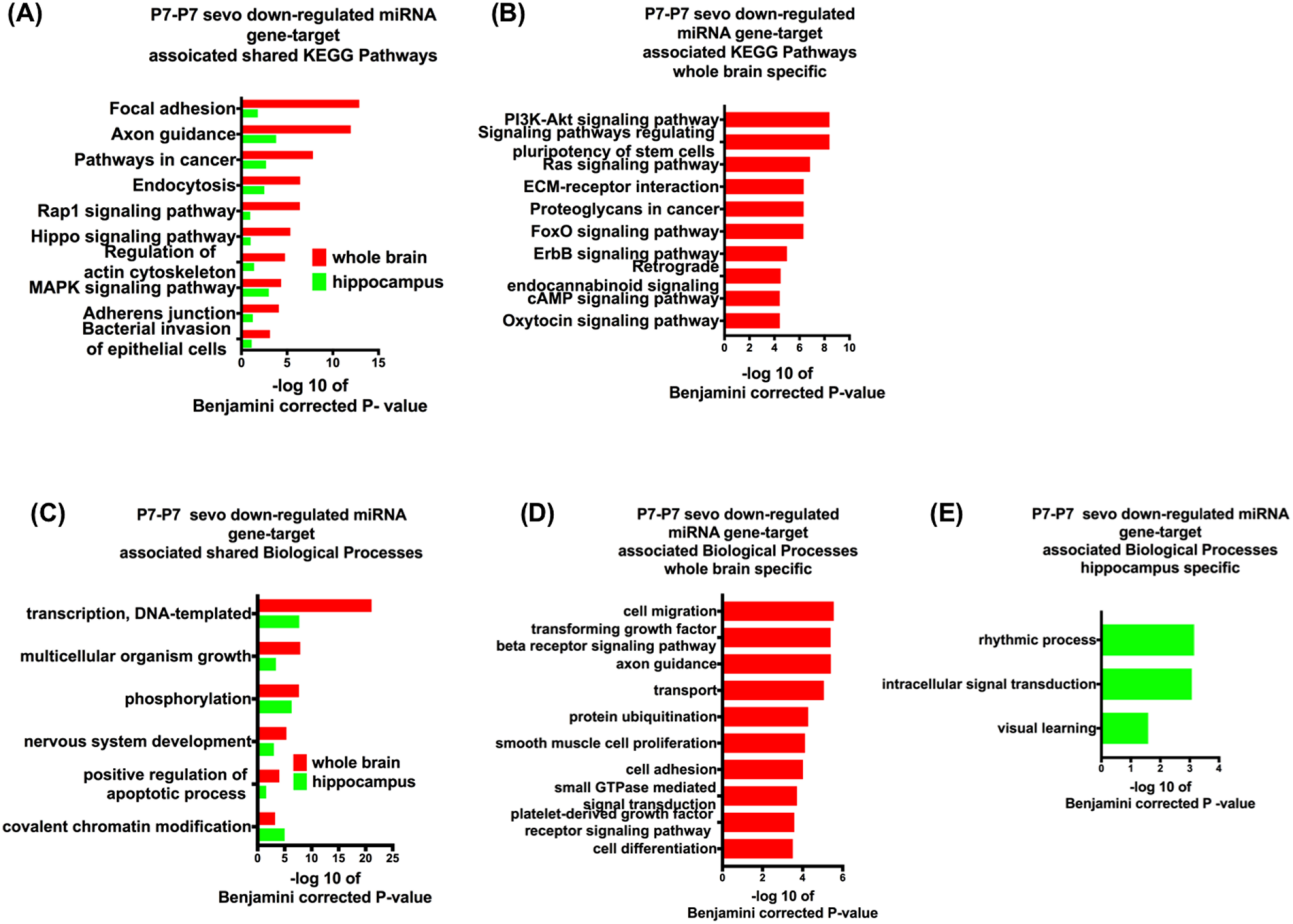
P7-P7 sevo down-regulated miRNA gene target associated KEGG pathway and biological process. P7-P7 sevo down-regulated miRNA gene- targets have shared (**A** and **C**), whole brain specific (**B** and **D**) KEGG pathways and biological processes and hippocampus specific (**E**) biological processes. (Pathways shown are based on Benjamini corrected p-value of <0.05, with only the top ten pathways shown. See Supplementary Figures for additional pathways within the analysis).

**Figure 5:**
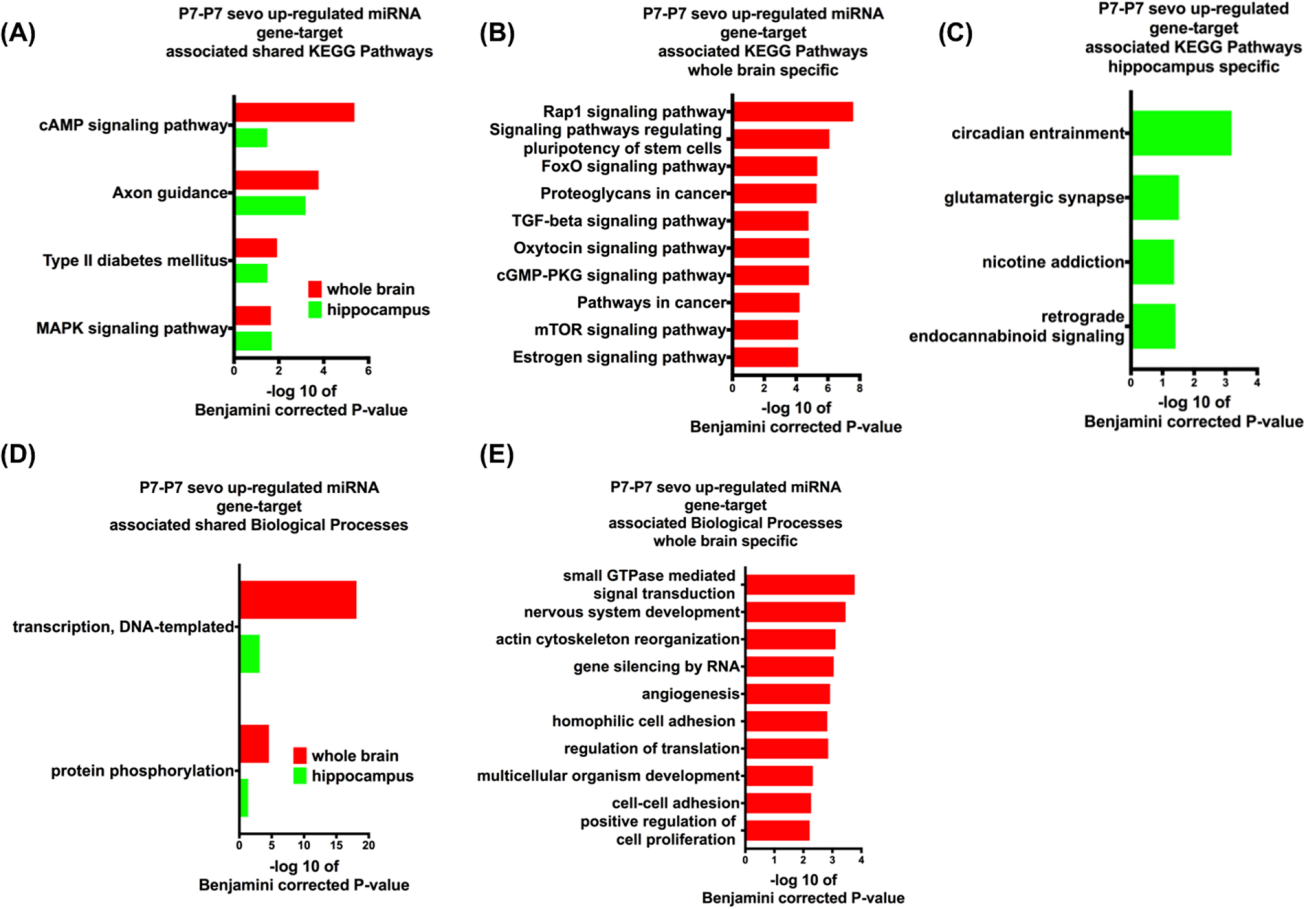
P7-P7 sevo up-regulated miRNA gene-target associated KEGG pathways and biological processes. P7-P7 sevo up-regulated miRNA gene targets have shared (**A** and **D**), whole brain specific (**B** and **E**) KEGG pathways and biological processes and hippocampus specific (**C**) KEGG pathways. (Pathways shown are based on Benjamini corrected p-value of <0.05, with only the top ten pathways shown. See Supplementary Figures for additional pathways within the analysis).

Based on Table 1, in the whole brain, a set of 6 miRNAs that were altered by sevo treatment regulates 2866 putative gene targets. Similarily, in the hippocampus, a different set of 6 miRNAs altered by sevo treatment regulates 2410 putative gene targets. Could these miRNA gene targets from Table 1 be responsible for early-life sevo associated social and cognition behavior impairments later on in life? To understand the functional significance of these miRNA gene targets, three groups of bioinformatics analyses were employed. The first group consisted of all the miRNAs of Table 1 as the P7-P7 sevo-regulated miRNA gene targets (Fig. 3). The second group had only the P7-P7 sevo-down-regulated miRNA gene targets miR-136, 145, 29a, 300, 15a, 20, 382, 532-5 and 99a from Table 1 (Fig. 4) and the third group had only the P7-P7 sevo-upregulated miRNA gene targets miR-19a, 361 and 1.4 from Table 1 (Fig. 5). One striking observation is that, axon guidance, a process that is critical for nervous system development, is present in all three groups based on KEGG (Kyoto Encyclopedia of Genes and Genomes) pathway analysis (Figs 3A, 4A and 5A). The KEGG pathways is a compilation of manually verified pathways based on annotated genomes by displaying both the molecular interactions and the biochemical reactions^28^. Two molecular mechanisms, DNA transcription and protein phosphorylation are shared by all three Biological Process analyses (Figs 3D, 4C and 5D). Within each analysis, the whole brain and the hippocampus also have their own unique pathways, which are mostly associated with neurodevelopmental processes (Pathways shown are based on Benjamini corrected p-value of <0.05, with only the top ten pathways shown. See Supplementary Figures for additional pathways within these analyses).

### Neonatal sevo’s long-term impact on miRNA expression and its associated functional pathways

**Figure 6:**
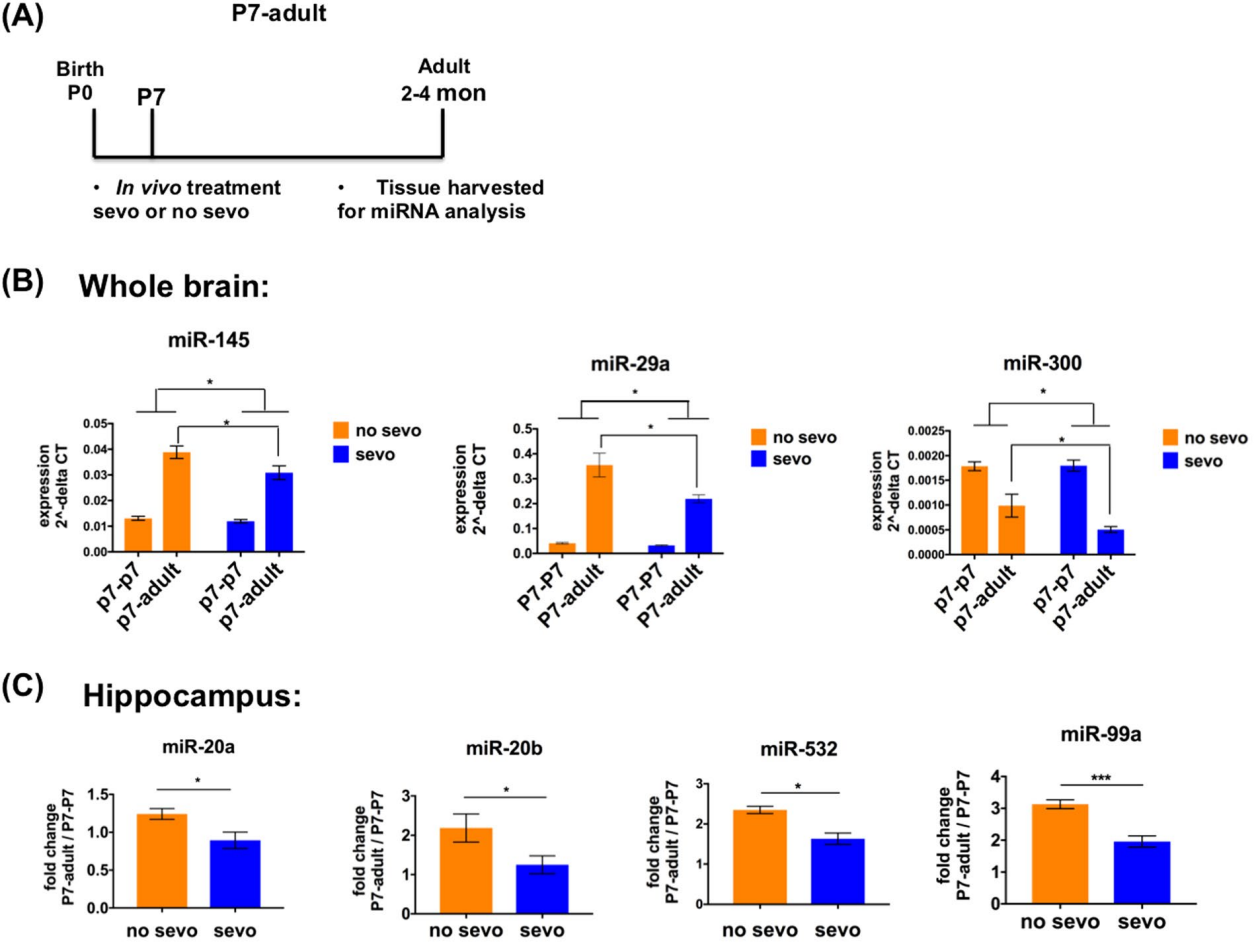
Neonatal sevo has a long-lasting effect on P7-P7 sevo-regulated miRNAs. (**A**) The P7-adult group represents mice that were treated at P7, followed by tissue collection when the mice were adults (**B**) In the whole brain, P7 sevo treatment has long-lasting effect on miR-145, miR29a, and miR300 (Two-way ANOVA, treatment, comparing no sevo from the combined P7-P7 and P7-adult vs. sevo from the combined P7-P7 and P7-adult, P < 0.05; posthoc, no sevo vs. sevo in the P7-adult, P < 0.05). (**C**) In the hippocampus, P7 sevo treatment has long-lasting effect on miR-20a, 20b, 532 and 99a. This effect was observed through the expression fold change, P7-adult/P7-P7 (t-test, no sevo vs. sevo, P < 0.05). * denotes P < 0.05.

In addition to P7 sevo treatment’s immediate impact on specific miRNAs, we wondered first, if such impact is transient or long-lasting and second, whether the expression of these miRNAs has an developmental component? To address these questions, we used real-time PCR to examine the miRNAs from Table 1 in a new group of samples, P7 treated - adult tissue harvested (P7-adult) (Fig. 6A). We noticed that among tissue from the whole brain, P7 sevo treatment had a long-lasting significant effect on the expression of miR-145, miR-29a and miR-300. Specifically, for the P7-adult group, the expression of the no sevo group is significantly different from the sevo group (Two-way ANOVA, treatment effect, comparing all no sevo to all sevo groups, *denotes, P < 0.05. Followed by posthoc, P < 0.05 comparing no sevo vs. sevo of the P7-adult group) (Fig. 6B). In the hippocampus, P7 sevo treatment’s long-lasting effect was observed in miR-20a, miR-20b, miR-532 and miR-99a based on fold change from the P7-P7 to the P7-adult (P7-adult / P7-P7) (Unpaired t-test, * denotes P < 0.05; ** denotes P < 0.01). The data demonstrate that, neonatal sevo not only has immediate effects but also long-lasting effects on specific miRNAs from Table 1 that can be observed during adulthood.

However, significant fluctuation in expression as a result of age is not always associated with neonatal sevo’s long-lasting impact. There are miRNAs from Table 1 that did not show differential expression in P7-adult comparing no sevo vs. sevo, but showed significant fluctuation in expression as a result of age, such as miR-19a and 361 from the whole brain and miR-15a, 129-3p and 382 from the hippocampus (Supplementary Figure 5).

**Figure 7:**
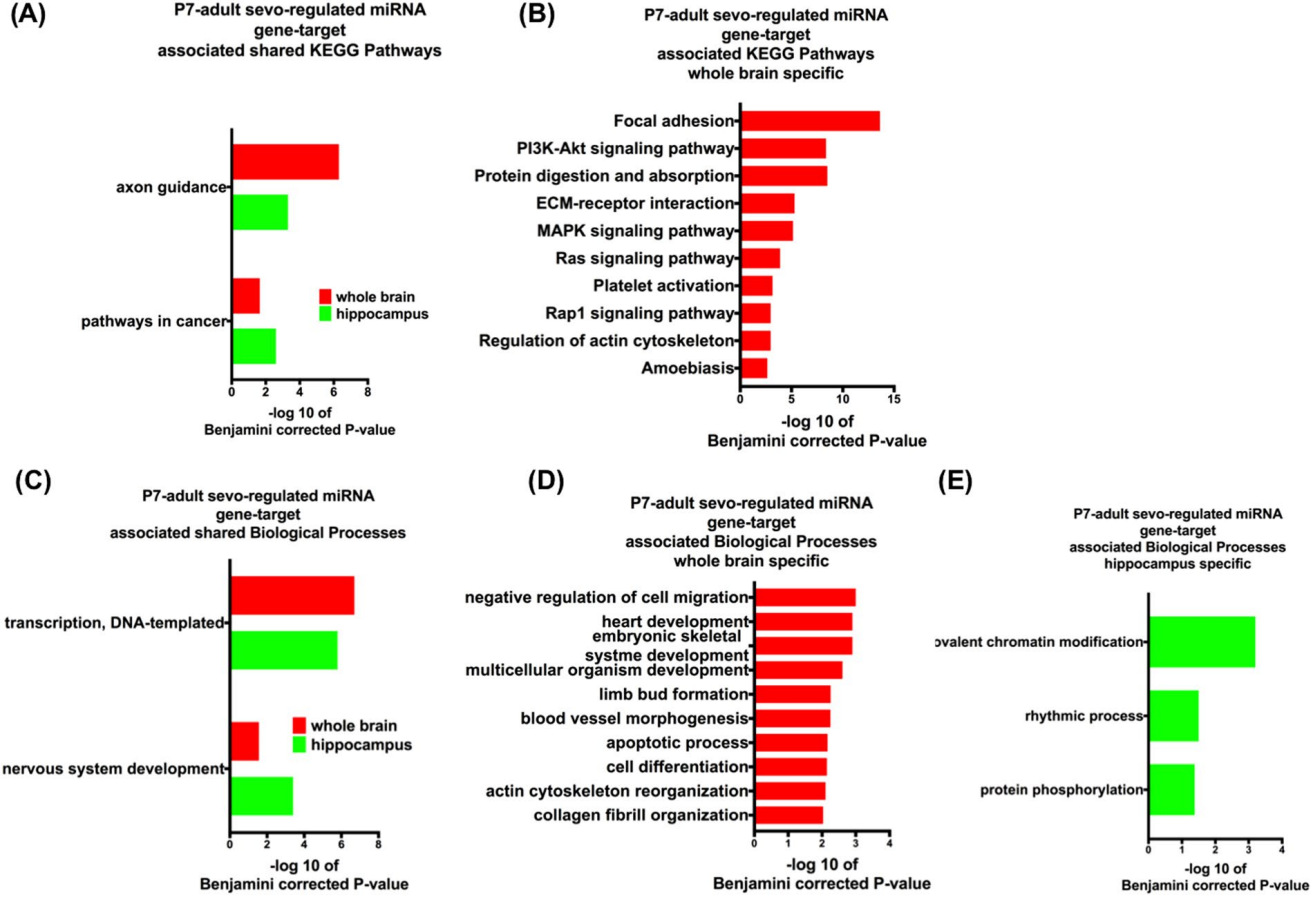
P7-adult sevo regulated miRNA gene-target associated KEGG pathways and biological processes. Sevo-regulated miRNA (from Fig. 6, identified miRNAs in the P7-adult group are all downregulated as a result of P7 sevo) gene-targets have shared KEGG pathways and biological processes between the whole brain and hippocampus (**A** and **C**). Whole brain is also shown to have its own specific (**B** and **D**) KEGG pathways and biological processes. (**E**) Hippocampus is shown to have its own specific biological processes. (Pathways shown are based on Benjamini corrected p-value of <0.05, with only the top ten pathways shown. See Supplementary Figures for additional pathways within the analysis).

We also examined the functional role of this set of miRNAs (Fig. 7) with long-lasting expression changes as a result of P7 sevo. Interestingly, axon guidance, DNA transcription and nervous system development continued to be enriched in KEGG pathway and Biological process analyses (Pathways shown are based on Benjamini corrected p-value of <0.05, with only the top ten pathways shown. See Supplementary Figures for additional pathways within the analysis). These data suggest the functional effect associated with neonatal sevo induced miRNA expression change is long-lasting.

**Figure 8:**
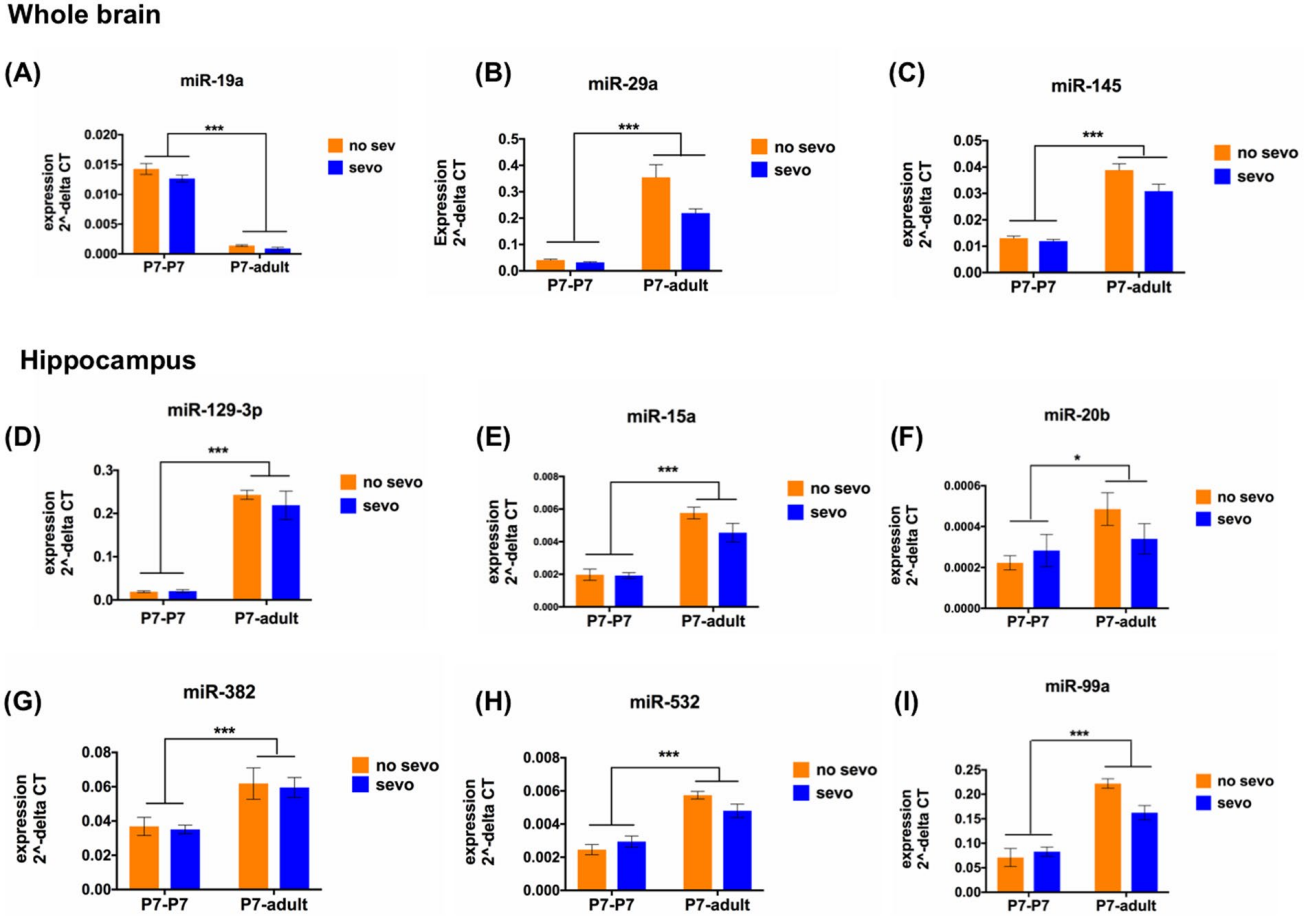
P7 sevo-regulated miRNA has temporal expression pattern. (**A**–**C**) In the whole brain, miR-145, 19a and 29a showed significant change in expression from P7-P7 to P7-adult. (**D**–**I**) In the hippocampus, P7 sevo regulated miRNAs, miR-129-5p, 15a, 20b, 382, 532 and 99a showed significant change in expression from P7-P7 to P7-adult (Two-way ANOVA for age: P7-P7, combining both no sevo and sevo vs. P7-adult, combining both no sevo and sevo, P < 0.001, for all identified miRNAs, expect for miR20b, which showed a P < 0.05). * denotes P < 0.05 and *** denotes P < 0.001.

Developmentally, all 6 miRNAs from the hippocampus (miR-15a, miR-20b, miR-382, miR-532, miR-129-3p and miR-99a) and 3 out of 6 miRNAs from the whole brain (miR-19a, miR-29a and miR-145) showed significant changes in expression from P7 (P7-P7, combining both no sevo and sevo) to adult (P7-adult, combining both no sevo and sevo) (Fig. 8). By using real-time PCR, we show that most expression change had an increased pattern as a result of age except for miRNA-19a, which was significantly decreased from P7-P7 to P7-adult (Two-way ANOVA, age effect, * denotes, P < 0.05; *** denotes, P < 0.001).

## Discussion

Converging evidence from the past decade of research has brought awareness among clinicians and the FDA of the potential risks associated with early-life anesthetic exposure^29^. However, for many young children with medical conditions, the need for surgery outweighs the risks of anesthetic exposure. Therefore, the most effective approach to resolve this emerging dilemma would be the discovery of therapeutic interventions, a process that can be facilitated by our understanding of epigenetic/molecular mechanisms, such as data from this study on the role of miRNAs underlying neonatal anesthetic neurotoxicity.

This is the first study that directly examines the *in vivo* effect of neonatal sevo exposure on the expression of brain miRNAs. Sevo’s main anesthetic property is to modulate the GABA_A_ receptor thereby enhancing its synaptic inhibitory transmission^30^. Since GABA_A_ receptors are ubiquitously expressed throughout the brain, we profiled miRNA expression using whole brain samples. In addition, we also examined the hippocampus based on our understanding of its association with neonatal sevo-associated learning and memory deficits, demonstrated previously by the hippocampus specific, active place avoidance task^26,31^.

miRNA expression from the profiling data is highly heterogeneous and a global change in miRNA expression was only observed when comparing the whole brain and the hippocampus, not when comparing no sevo and sevo (Fig. 2). There are several explanations for this observation. First, brain miRNAs are differentially expressed in distinct brain regions and neuronal cell types^32^. Second, like miRNAs from other tissues, expression of brain miRNAs is highly variable. For example, miR-9, one of the most highly expressed miRNAs in the developing and adult vertebrate brain^33^, is also shown to have the highest expression in our profiling data. While other known brain candidates, such as miR-190^34^, is minimally detectable (Fig. 1). Third, if neonatal sevo produced a global change in miRNA expression, sever malformations in the central nervous system would occur, such as those seen in Dicer-deficient zebrafish that failed to produce mature miRNAs^35^.

P7 sevo treatment had immediate (P7-P7 group) and long-lasting (P7-adult group) impact on the expression of specific miRNAs. These miRNA gene-targets have shared KEGG pathways relating to neuronal morphological development, such as focal adhesion, axon guidance and actin cytoskeleton (Figs 3, 4 and 5). These KEGG pathways are shared among the identified miRNAs from the whole brain and the hippocampus. Furthermore, the axon guidance pathway continues to be enriched in the P7-adult group. This is interesting because the number of differentially expressed miRNAs in the P7-adult group is reduced (Fig. 6) compared to the candidates initially identified in Table 1. These pathways are in agreement with studies that demonstrated neonatal sevo’s impact on axon guidance in developing mouse neocortical neurons^24^ and crucial elements of developmental synaptogenesis such as, actin cytoskeleton disorganization and impaired dendritic branching^36^. Another category that is significantly enriched in the KEGG pathway is signaling pathways that are mainly involved in cellular processes like proliferation, differentiation and stress. Evidence of neonatal sevo’s effect on signaling pathways is less clear, however, sevo-regulated miRNA gene targets are associated with apoptosis, as shown directly by our biological pathway analysis (Figs 3, 4, 5 and 7). This is in concert with numerous reports that showed neonatal anesthetic exposure is associated with neuronal apoptosis^9^. Mechanisms of apoptosis are regulated by signaling pathways such as Wnt, MAPK and Rap1 (pathways identified and listed on shared KEGG pathways), which support an association between neonatal sevo-associated miRNAs and signaling pathways.

In addition to bioinformatic evidence on neonatal sevo-assoicated miRNAs in neurodevelopment, some of these miRNAs have been shown to have a direct functional role in the brain. A few examples are, miR-29 modulates axonal branching by regulating the protein doublecortin^37^; miR-19 is critical for irregular migration of newborn neurons in the adult brain that may contribute to the etiology of schizophrenia^38^; miR-382 from the nucleus accumbens is associated with alcohol addiction^39^; and miR-99a has neuroprotective effects by regulating cell cycle progression and preventing apoptosis^40^.

miRNA expression in the brain is known to have a developmental component. For example, miRNAs in human postmortem brains showed differential expression comparing ages from infancy, to early childhood, late childhood and adolescence^41^. These differentially expressed miRNAs targets were highly enriched for gene sets related to autism, schizophrenia, bipolar disorder and depression. This evidence suggests that miRNAs with developmentally differential expression are critically associated with neurodevelopmental disorders. Similarly, comparing the time points from groups P7-P7 to P7-adult, we observed a significant expression change in neonatal sevo-associated miRNA. Putative gene targets under the control of these specific miRNAs are mostly related to the processes of transcription regulation and nervous system development.

Our evidence of neonatal sevo’s immediate and long-lasting effects on specific miRNAs and their associated neurodevelopmental functional pathways highlights the central function of these miRNAs in shaping the behavioral changes that we observed in adulthood. With emerging roles of miRNAs in perioperative medicine^42^, designing pharmacologic approaches to modulate miRNA functions would be a potential therapeutic approach for neonatal anesthetic related neurotoxicity.

## Material and Methods

### Treatment with sevoflurane

C57/BL6 mice were used throughout the study, which was approved by the SUNY Downstate IACUC. All experiments were performed in accordance with relevant guidelines and regulations. At postnatal day 7 (P7), male pups from each litter (ranging from 2–6 pups) were randomly assigned to either the sevo or the no sevo (control) treatment group, while the female pups remained with the dam, as described in our previously established protocol^26^. During a 2-hour treatment period, pups from the sevo group were separated from the dam and exposed to 2.4% sevo in a 40% oxygen (O_2_) 60% nitrogen (N_2_) gas mixture (GTS-WELCO, Newark Distribution). These pups were placed on a 37 °C heating pad to prevent hypothermia during treatment. A pulse oximeter sensor (MSTAT-4mm, Kent Scientific Corporations) was placed on one of the hind paws of the pup and measurements of heart rate and blood oxygen saturation (SPO_2_) were recorded every five minutes. The sevo concentration, peripheral capillary oxygen saturation (SpO_2_) and heart rate (HR) were recorded every 5 minutes. The pups from the no sevo group were also separated from the dam and exposed only to 40% O_2_ and 60% N_2_.

### Brain tissue

Mice that underwent P7 sevo or no sevo treatment were separated into two groups: the P7-P7 and the P7-adult group. For the P7-P7 group, at the end of the 2-hour treatment, pups were sacrificed and brain tissues were harvested immediately. For the P7-adult group, at the end of the 2-hour treatment, the pups were returned to their home cage and reunited with their dams to be reared and weaned following standard institution procedures. The brains from the P7-adult group were then harvested when the mice have reached 2–4 months of age. For tissue collection, the mouse was sacrificed by cervical dislocation, followed by decapitation and brain removal. For whole brain samples, the brain was removed in its entirety. For hippocampus sample collection, the brain was removed, followed by dissection and retrieval of the hippocampus.

### miRNA profiling and expression analysis

For miRNA profiling, samples were collected from the P7-P7 group (N = 3 for each of the whole brain and the hippocampus group). Total RNA was isolated from either the whole brain or the hippocampus by using miRNeasy Mini (Qiagen). miRNA profiling was examined based on the known 599 rodent miRNAs by using the nCounter miRNA Expression Assay (Nanostring Technologies). Real-time PCR was used to further analyze the expression of specific miRNAs for the P7-P7 and the P7-adult groups. These are not the same samples used for the profiling study, rather, separate groups of mice were treated and samples prepared (N = 5–7 for each group). Small RNAs from the samples were isolated by mirVana miRNA Isolation Kit (Life Technologies). cDNA of the small RNAs were reverse transcribed by TaqMan MicroRNA Reverse Transcription Kit. The Real-time PCR reaction was set up according to the TaqMan Small RNA Assays (Applied Biosystems) and assayed by CFX Connect Real-Time PCR Detection System (Bio-Rad).

### KEGG Pathway and Biological Process Analysis

Bioinformatics programs were used to obtain specific miRNA-associated gene- targets (Targetscan, MIT, 7.1). Each specific miRNA yielded a list of gene-targets. For the P7-P7 group, 3 lists were compiled to form either the neonatal sevo-regulated, sevo-downregulated or the sevo-upregulated list. All the miRNA-associated gene-targets on each list were subsequently used in to identify the associated KEGG Pathways and Biological Processes^43,44^ (David Database 6.7). Only pathways with Benjamini corrected p-value of P < 0.05 were used in our data analysis.

### Statistical analysis

Statistical analysis was done using GraphPad Prism 7.0.

### Data availability

The datasets generated or analyzed during this study are included in this published article (and its Supplementary information files).

## Electronic supplementary material

Supplementary figures and legends
