# Supplementary material for: Neonatal anesthesia exposure impacts brain microRNAs and their associated neurodevelopmental processes
Source: Sci Rep. 2018 Jul 13;8:10656. doi: 10.1038/s41598-018-28874-0 (PMC6045579; doi:10.1038/s41598-018-28874-0)
Supplement: Supplementary file 1 — Supplementary figures and legends [file 41598_2018_28874_MOESM1_ESM.pdf]

Supplementary Information

Neonatal anesthesia exposure impacts brain microRNAs and their associated neurodevelopmental processes

Daisy Lin[1,2 *], Jinyang Liu[1], Zihua Hu[3], James Cottrell[1]
and Ira S. Kass[1,2]

Anesthesiology Department[1], The Robert F. Furchgott Center for Neural and Behavioral Sciences, Department of Physiology and Pharmacology[2],
SUNY Downstate Medical Center, and Center for Computational Research, New York State Center of Excellence in Bioinformatics & Life Sciences[3], SUNY Buffalo.

Daisy Lin*
Anesthesiology Department
SUNY Downstate Medical Center, Box 6
450 Clarkson Ave.
Brooklyn, NY 11203
Email: daisy.lin@downstate.edu
Phone: 718-270-2048, 718-270-1709

Supplementary

Figure 1.

(A)

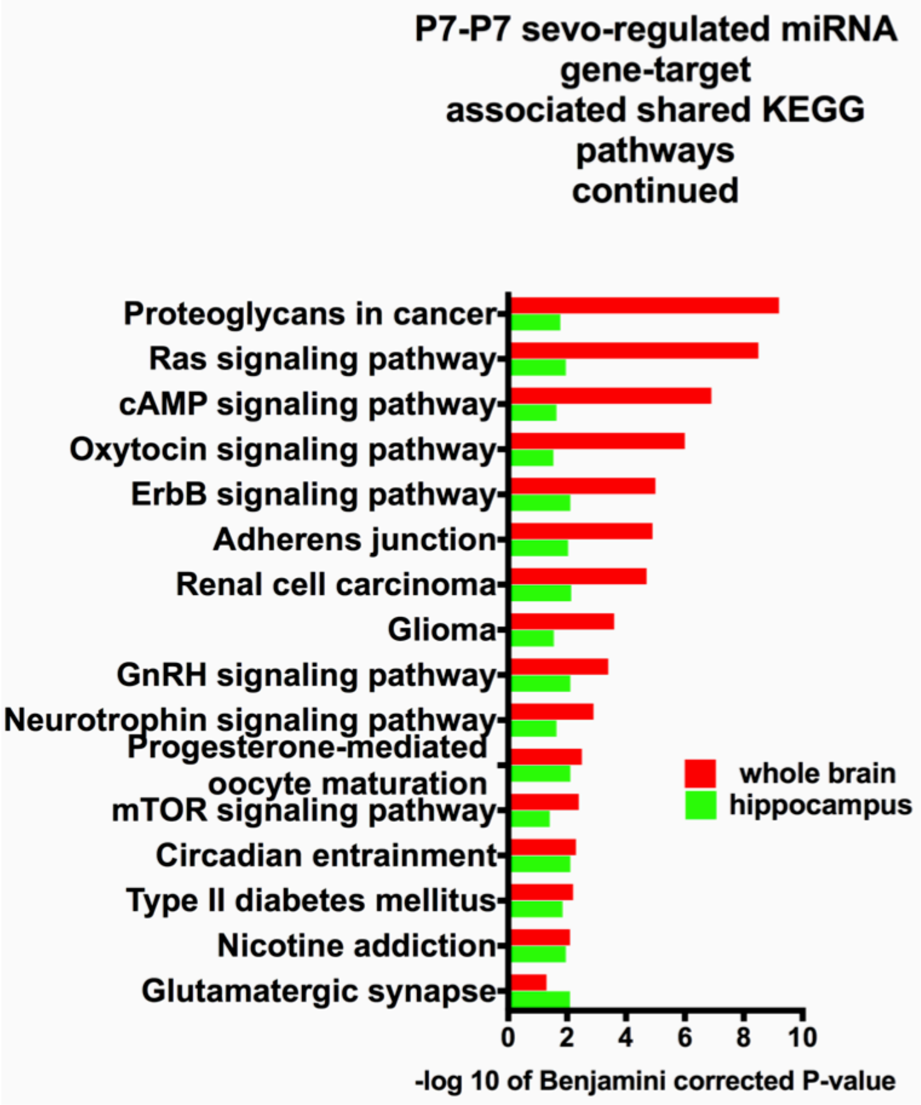

Supplementary Figure 1.

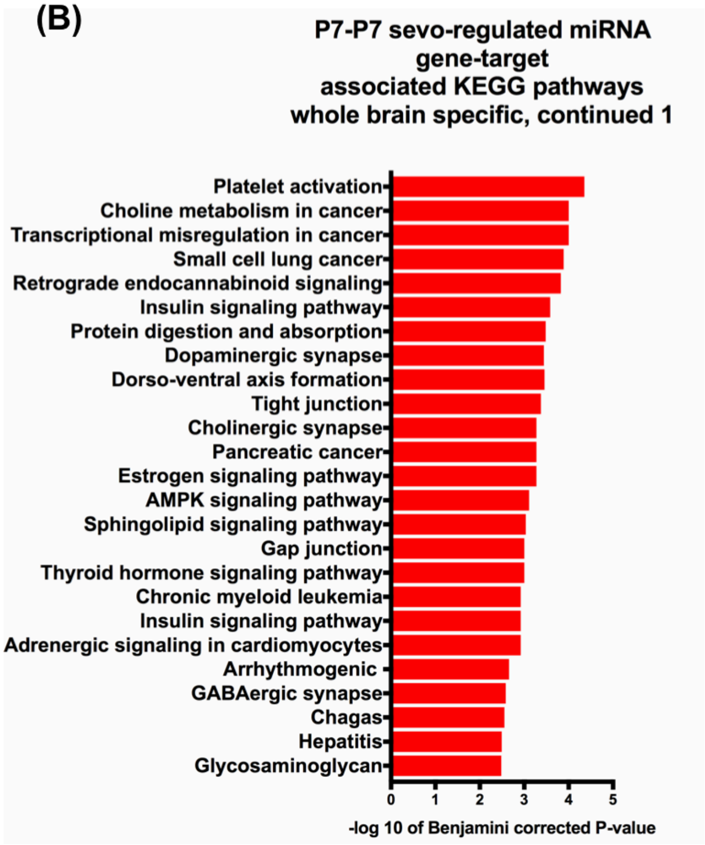

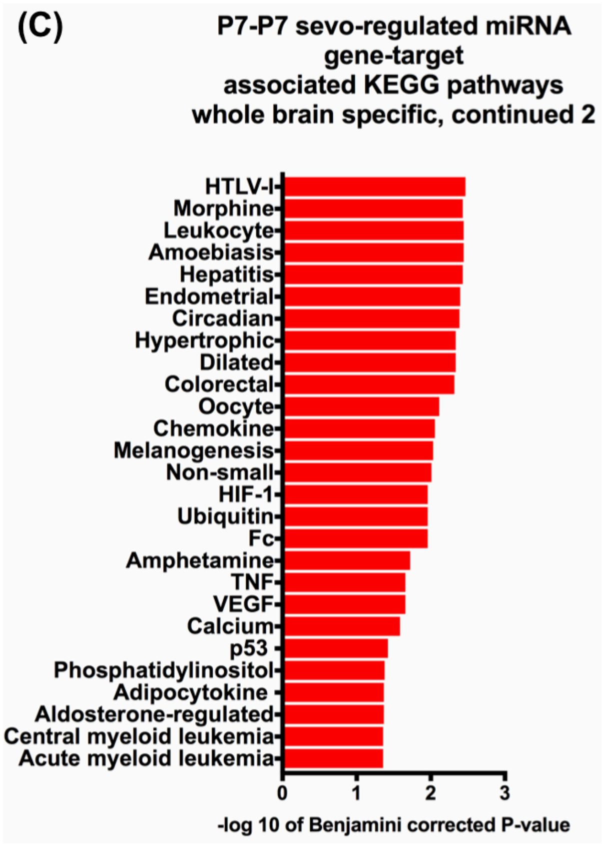

Supplementary Figure 1
(D)

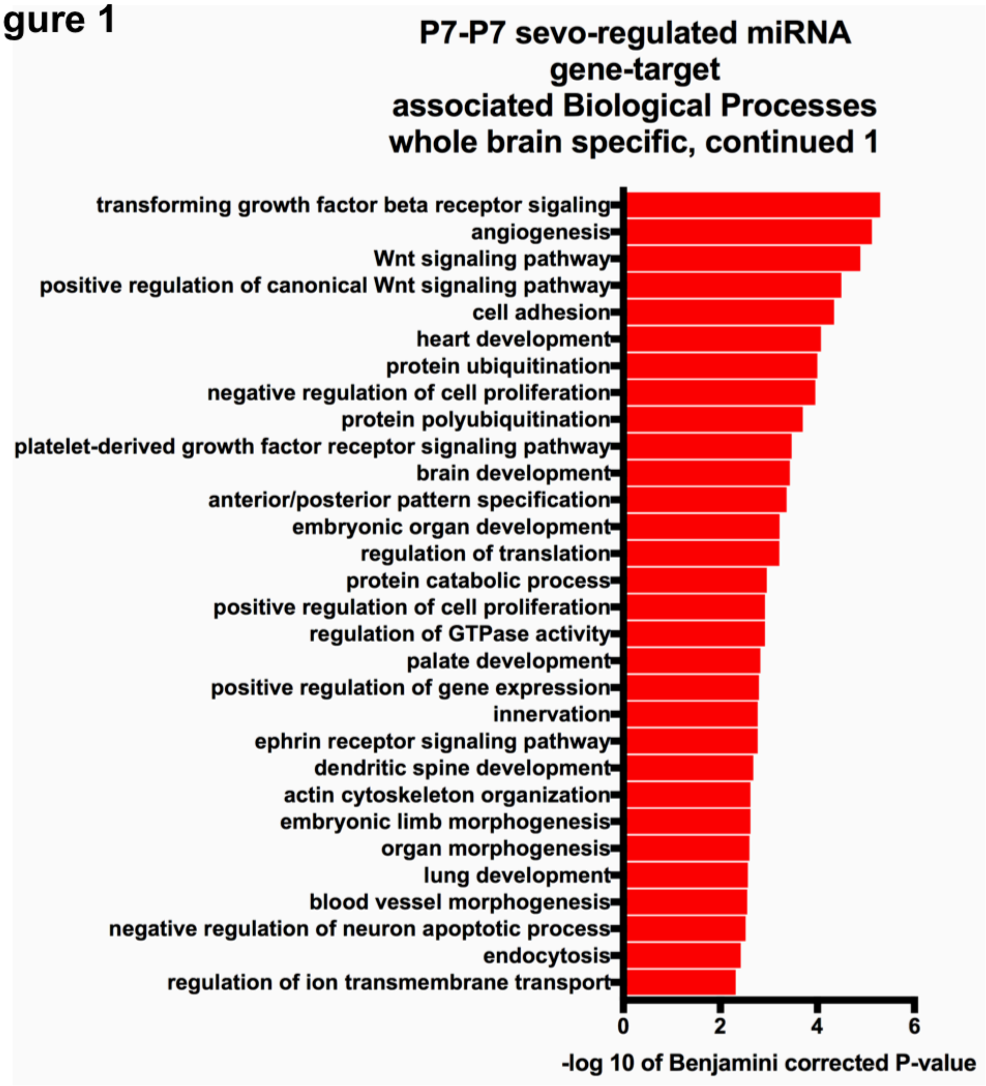

Supplementary Figure 1.
(E)

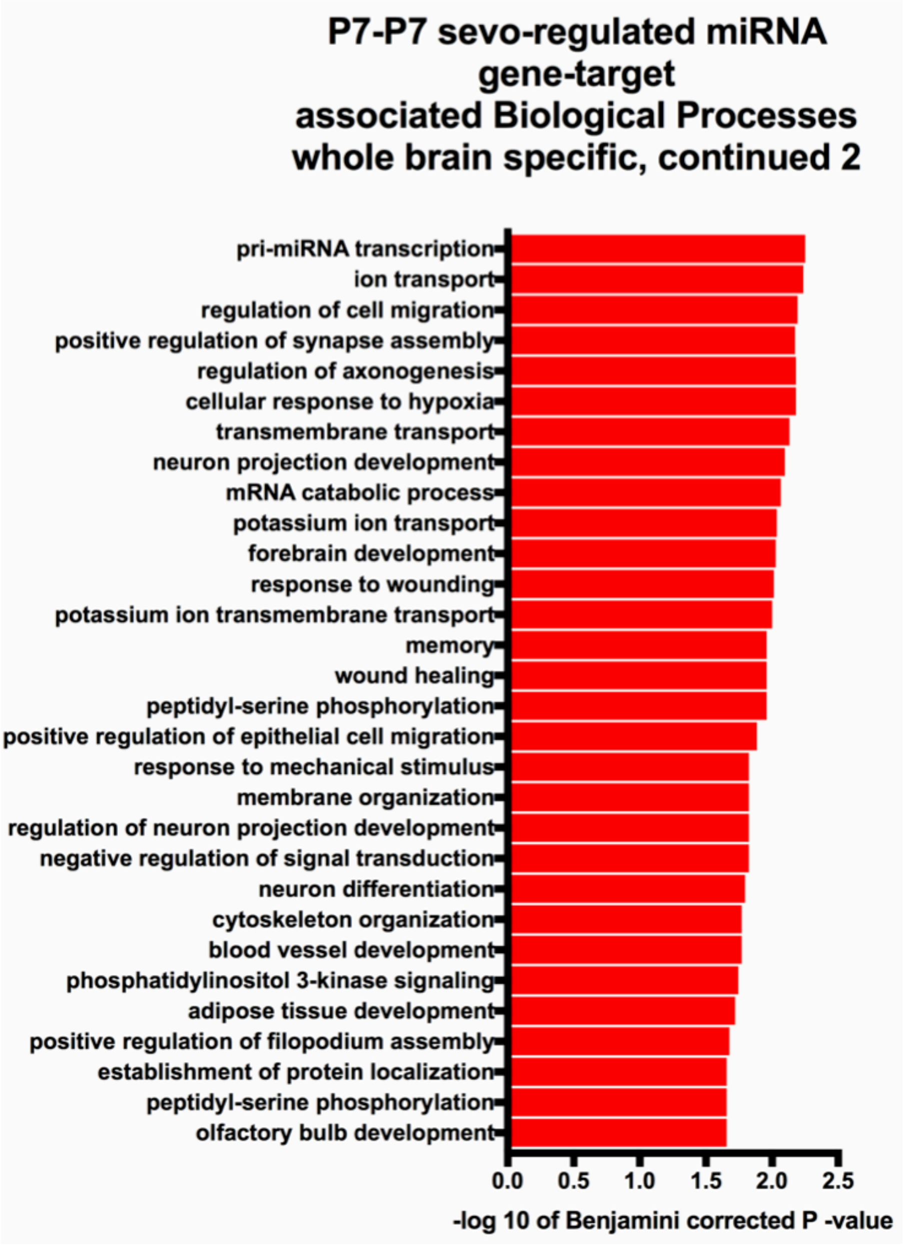

Supplementary Figure 1.
(F)

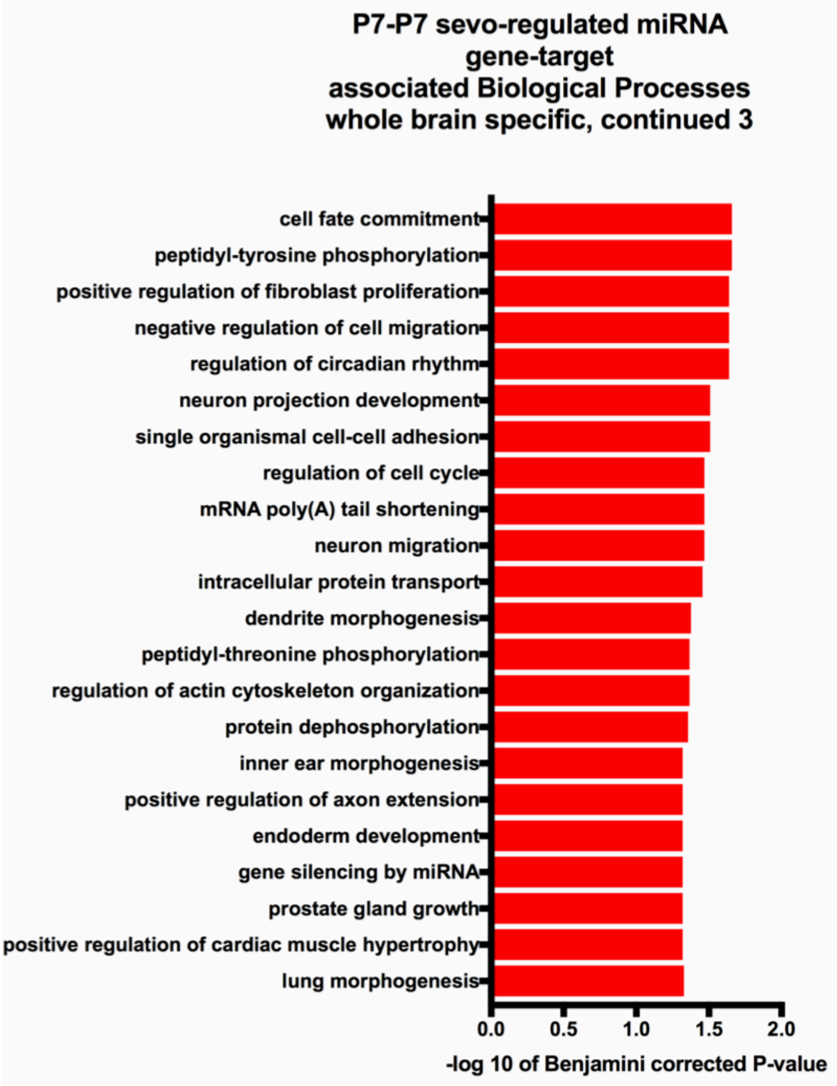

Supplementary Figure 2.
(A)

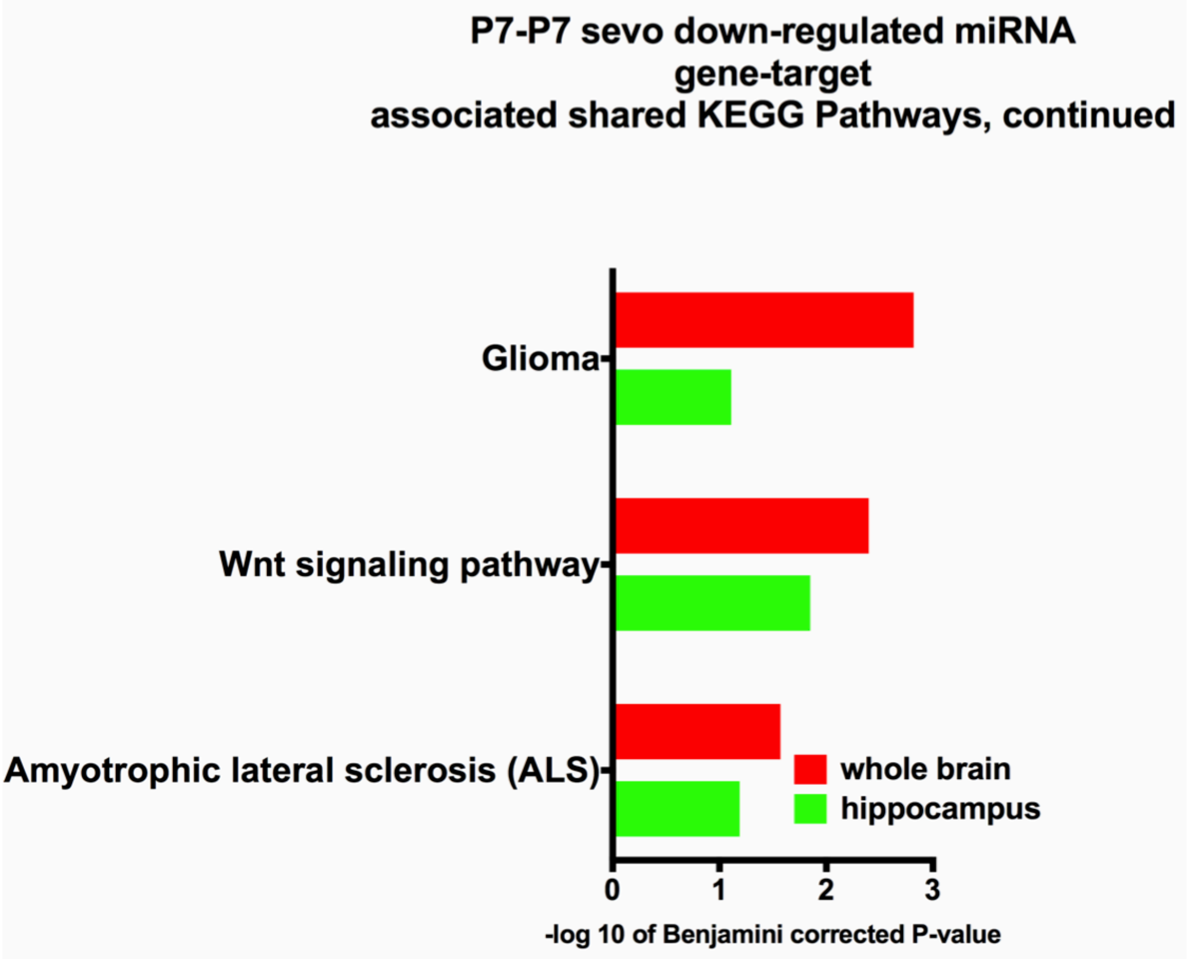

Supplementary Figure 2.

(B)

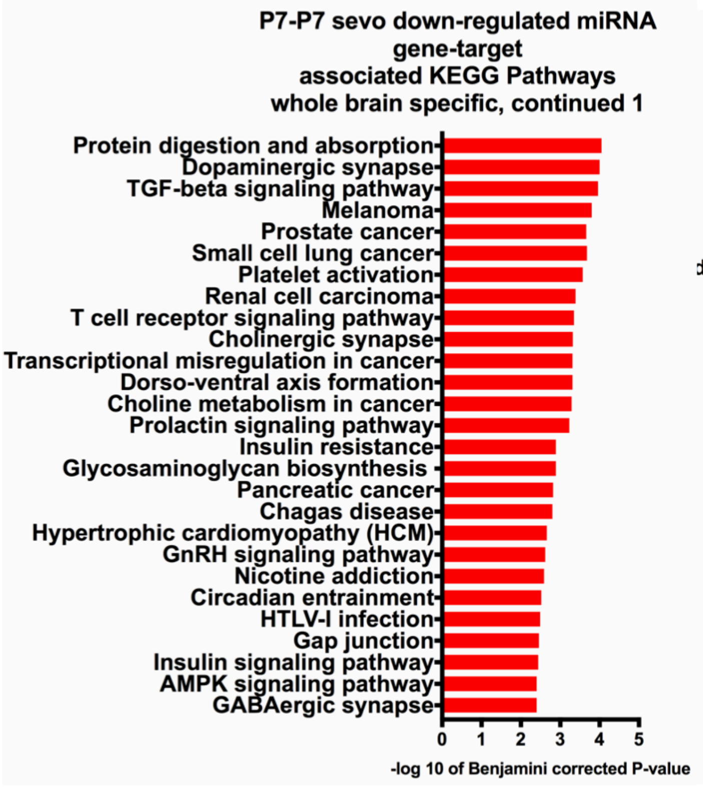

(C)

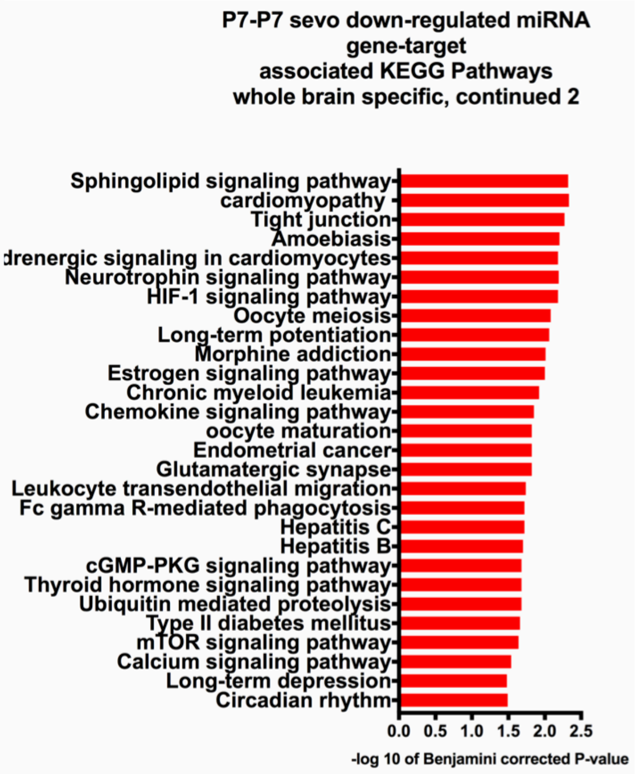

Supplementary Figure 2.

(D)

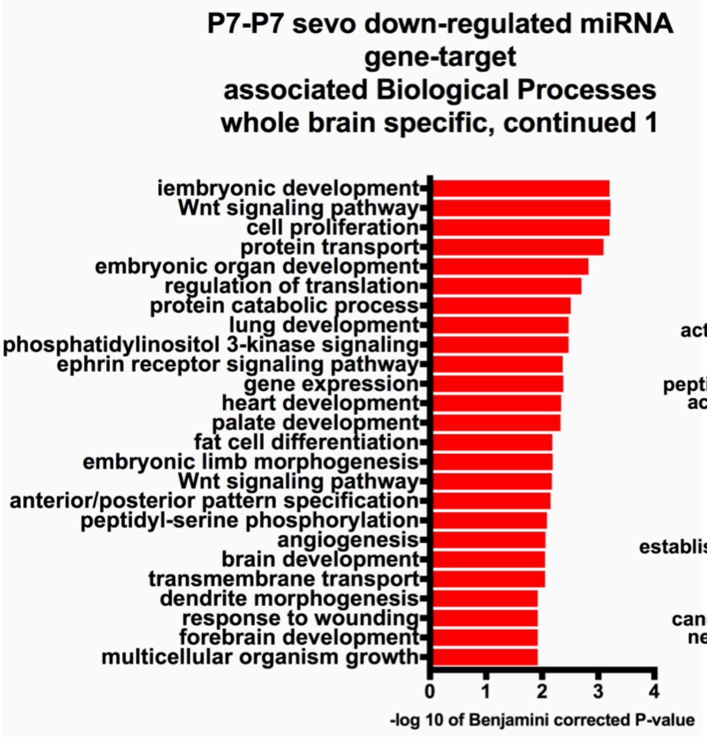

(E)

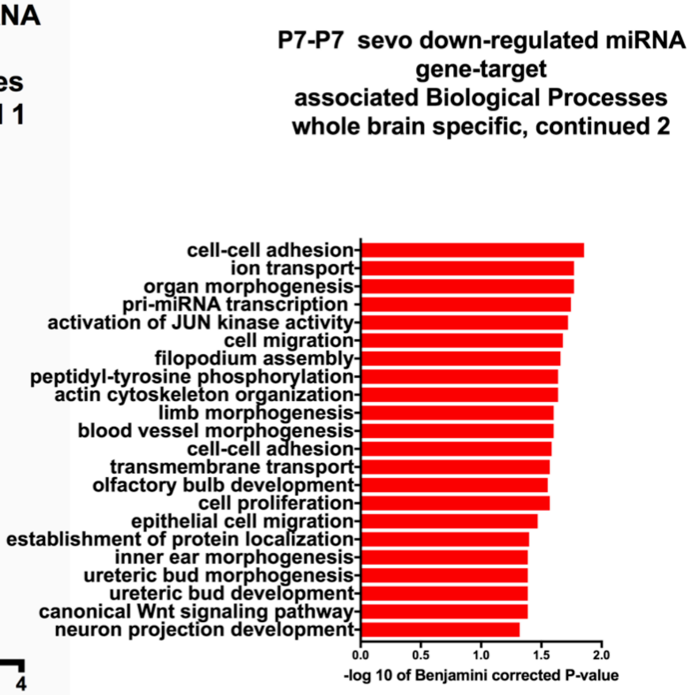

Supplementary Figure 3.

(A)

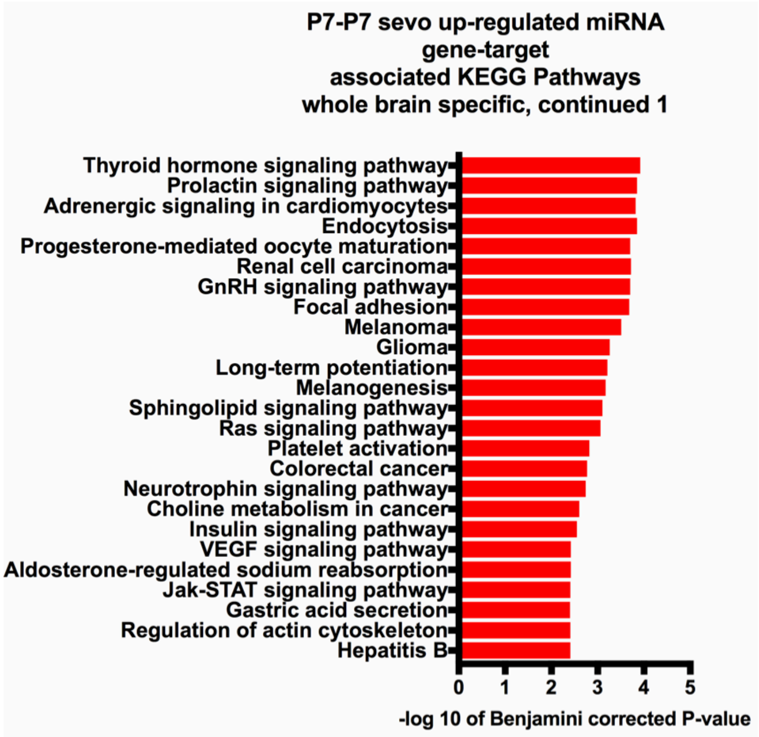

(B)

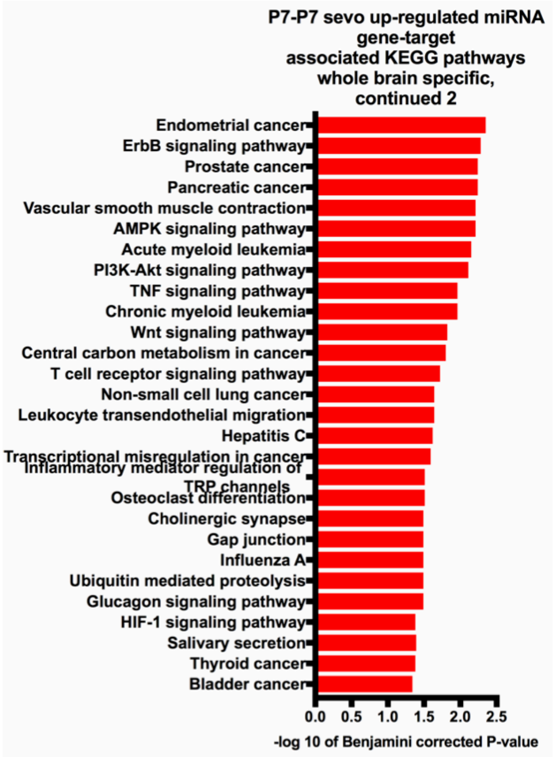

Supplementary Figure 3.
(C)

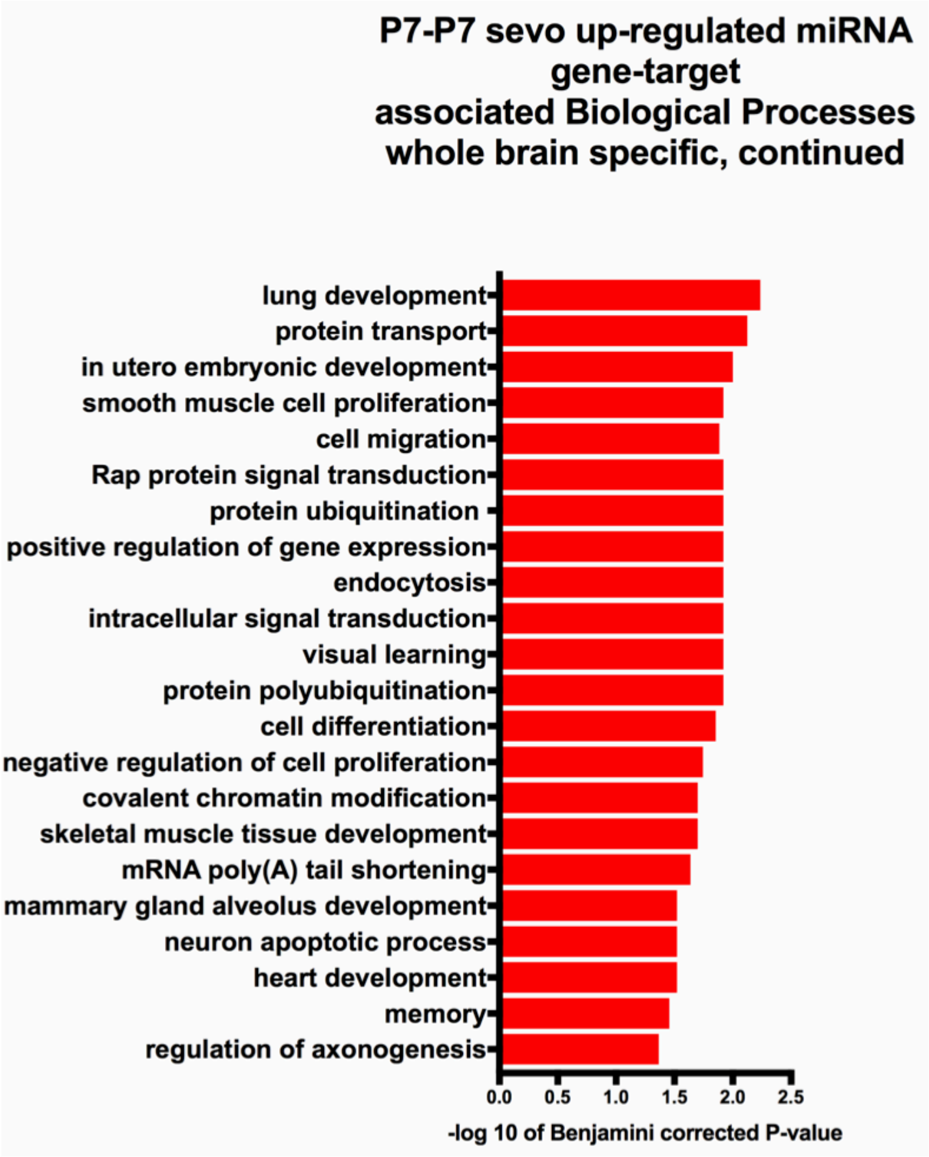

Supplementary Figure 4.

(A)

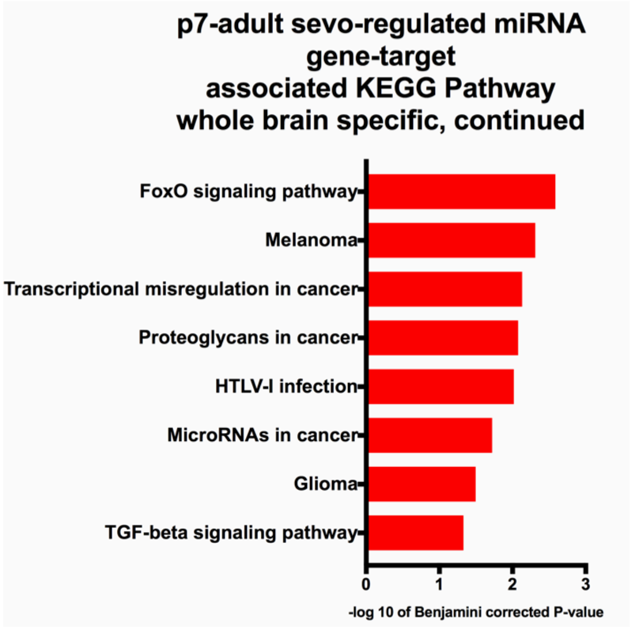

(B)

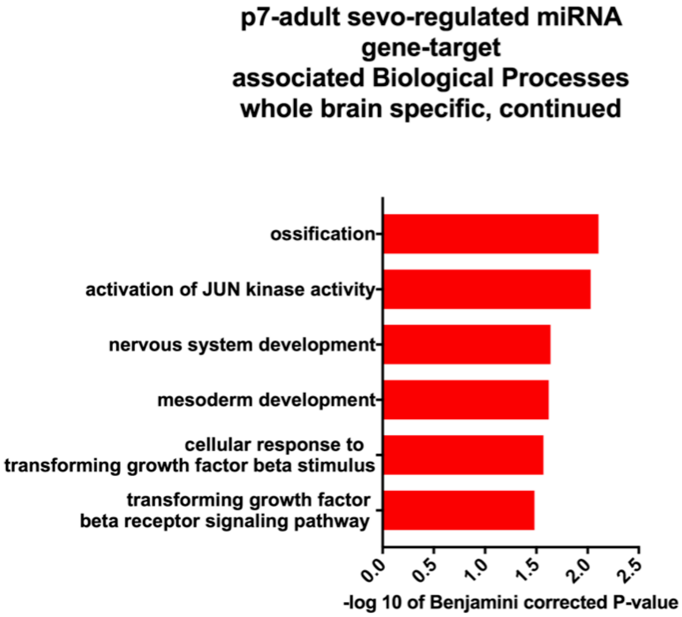

Supplementary Figure 5.

(A) Whole brain:

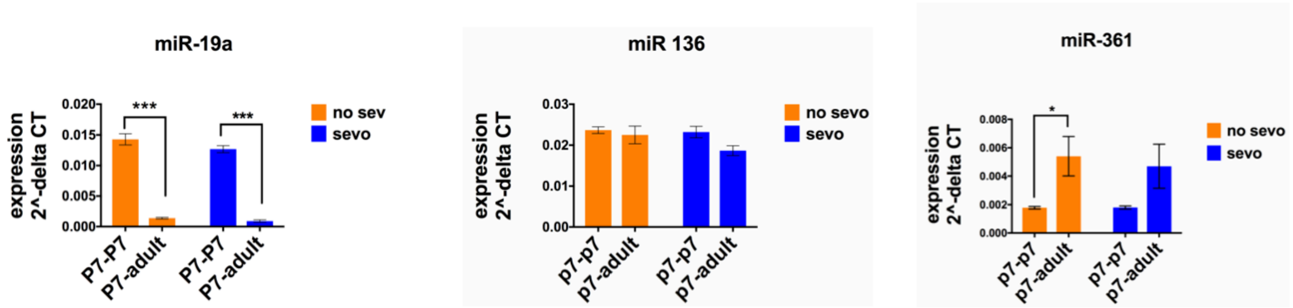

(B) Hippocampus:

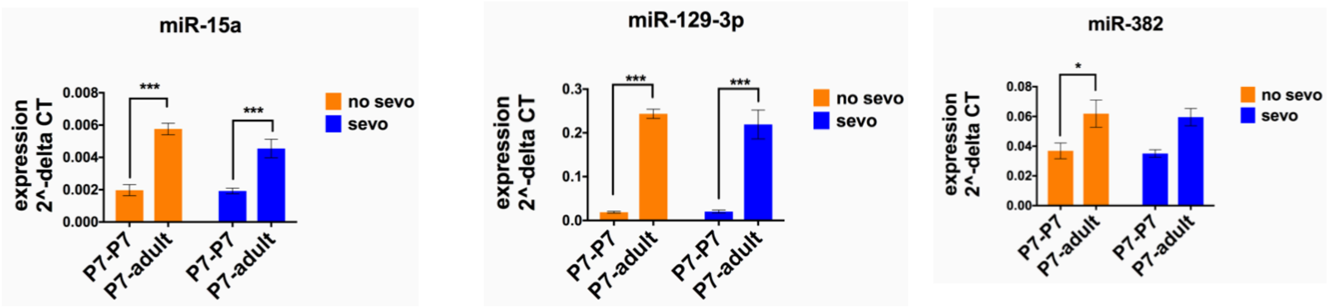

Supplementary Figures:

1) P7-P7 sevo-regulated miRNA gene-target associated KEGG pathways and Biological processes, continued after the top ten pathways. P7-P7 sevo-regulated miRNA (from Table 1, both up and down-regulated miRNAs) gene-targets have shared (A), whole brain specific (B, C, D and E) (Kegg pathways and biological processes. (Pathways shown are based on Benjamini corrected p-value of<0.05).

2) P7-P7 sevo down-regulated miRNA gene target associated KEGG pathway and biological process, continued after the top ten pathways. P7-P7 sevo down-regulated miRNA gene- targets have shared (A), whole brain specific (B, C, D and E) Kegg pathways

and biological processes. (Pathways shown are based on Benjamini corrected p-value of<0.05).

3) P7-P7 sevo up-regulated miRNA gene-target associated KEGG pathways and biological processes, continued after the top ten pathways. P7-P7 sevo up-regulated whole brain specific (A, B and C) Kegg pathways and biological processes. (Pathways shown are based on Benjamini corrected p-value of<0.05).

4) P7-adult sevo regulated miRNA gene-target associated KEGG pathways and biological processes, continued after the top ten pathways. Sevo-regulated miRNA gene-targets have whole brain specific (A and B) Kegg pathways and biological processes. (Pathways shown are based on Benjamini corrected p-value of<0.05).

5) P7 sevo treatment did not have long-lasting effect on the expression of the following miRNAs from Table 1. miR-19a, 136 and 361 from the whole brain and miR-15a, 129-3p and 382 from the hippocampus are not differentially expressed in P7-adult comparing no sevo vs. sevo. Among these miRNAs however, some are shown to have significant fluctuation in expression as result of age (P7-P7 vs. P7-adult) (* and *** denote P<0.05 and P<0.0001, respectively).
